# Screening for Cystic Fibrosis Related Complications in the Context of a Pandemic and Altered Models of Care

**DOI:** 10.1177/11786329231205145

**Published:** 2023-10-16

**Authors:** Michael Doumit, Roxanne Strachan, Raynuka Lazarus, Peter Middleton, Ruth Dentice, Jessica Marouvo, Laura Jeffrey, Hiran Selvadurai, Sheila Sivam, Verity Pacey, Adam Jaffe, Kelly Gray

**Affiliations:** 1 Department of Health Sciences, Macquarie University, Sydney, NSW, Australia; 2 School of Women’s and Children’s Health, University of New South Wales, Sydney, NSW, Australia; 3 Respiratory Medicine Department, Sydney Children’s Hospital, Sydney, NSW, Australia; 4 Respiratory Medicine Department, Westmead Hospital, Sydney, NSW, Australia; 5 Westmead Clinical School, University of Sydney, Sydney, NSW, Australia; 6 The Department of Respiratory Medicine, Royal Prince Alfred Hospital, Sydney, NSW, Australia; 7 Respiratory Medicine Department, The Children’s Hospital at Westmead, Sydney, NSW, Australia; 8 Discipline of Adolescent and Child Health, University of Sydney, Sydney, NSW, Australia; 9 Faculty of Medicine and Health, University of Sydney, Sydney, NSW, Australia

**Keywords:** Cystic fibrosis, variations in care, standards of care, outcome and process assessment, clinical audit

## Abstract

**Background:**

Standard of care recommend that patients with cystic fibrosis (CF) require screening investigations to assess for complications. Changing models of care due to the COVID19 pandemic may have impacted completion of recommended screening.

**Objective:**

To compare the frequency of screening investigations completed in people with CF before and after the onset of the COVID19 pandemic.

**Methods:**

Medical records were reviewed at 4 CF-specialist centers to identify screening investigations completed in the 12-months before and after pandemic onset.

**Results:**

Records of 625 patients were reviewed. Prior to pandemic onset, there was between center variability in completion of screening investigations. There was greatest baseline variation between centers in performing oral glucose tolerance test (OGTT); range 38%-69%, exercise tests; 3%-51% and sputum screening for non-tuberculous mycobacteria; 53%-81%. Following pandemic onset, blood tests, and sputum cultures were maintained at the highest rates. Exercise testing, CXR and OGTT exhibited the greatest declines, with reductions at individual centers ranging between 10%-24%, 22%-43%, and 20%-26%, respectively. Return to in-person visits following pandemic onset was variable, ranging from 16% to 74% between centers.

**Conclusion:**

Completion of screening investigations varies between CF centers and changes in models of care, such as increased virtual care in response to COVID19 pandemic was associated with reduction in completion of investigations. Centers would benefit from auditing their adherence to standards of care, particularly considering recent changes in care delivery.

## Introduction

Cystic fibrosis (CF) is a progressive, multi-system disease that requires lifetime monitoring and treatment. Comprehensive care includes inpatient and outpatient management which is primarily coordinated through CF specialist centers (CFSCs), as care delivered through CFSCs is associated with improved clinical outcomes.^1^ Comprehensive care also includes following international guidelines and adhering to recommended national standards of care,^2-4^ including recommendations that people with CF have certain investigations to screen for complications and monitor disease progression.

Early detection of CF related complications is an important step in early intervention as well as preventative care. For example, early intervention informed by the establishment of annual screening for cystic fibrosis related diabetes (CFRD) using oral glucose tolerance testing (OGTT) has been linked to improvements in nutritional status, lung function and mortality.^5^ Likewise, annual blood tests to detect abnormalities in vitamin levels and liver function have led to management changes before clinical manifestations occur.^6^ In addition, dual energy X-ray absorptiometry (DEXA) can detect early reductions in bone mineral density and inform interventions which may optimize bone health.^7,8^

Annual investigations are traditionally performed at CFSCs, however, CFSCs utilize different models of care when completing recommended annual screening investigations. Two such approaches are a dedicated annual review with all investigations completed at one point, compared to a continuous assessment approach, where investigations are spread throughout the course of a year.^9,10^ Changing the model of care from continuous assessment to a dedicated annual visit has been shown to increase completion of recommended investigations to more closely meet the recommended standards of care.^11^

The onset of the COVID19 pandemic led to urgent and necessary changes in models of care delivered to people with CF, with a dramatic reduction of in-person visits and a coinciding increase in use of telehealth.^12^ Australia wide, the percentage of clinical reviews conducted for people with CF via telehealth increased from 8% in the 12 months prior to pandemic onset to 47% in the 12 months following the pandemic onset, with an associated decrease in in-person attendance.^13^ This change in model of care which resulted in less frequent attendance to the CFSC may have impacted the frequency of investigations taking place.

Therefore, the aim of this study was to compare the frequency of recommended annual screening investigations taking place in people with CF in the 12 months before and after the onset of the COVID19 pandemic.

## Methods

### Design, study population and data source

This study was a retrospective medical record review of patients who attended 1 of 4 participating CFSCs in Sydney, Australia. Records were reviewed at 2 pediatric (Sydney Children’s Hospital, The Children’s Hospital at Westmead) and 2 adult CFSCs (Royal Prince Alfred Hospital and Westmead Hospital). Patients who had received their care at a single CFSC for the duration of the study period were included in the analysis. Patients were excluded if they were born, were deceased, underwent transplant or moved care to a different CFSC within the study period.

Data were obtained for time periods before and after the onset of the COVID-19 pandemic in Australia. The 16th March 2020 was chosen as the date from which the impact of the pandemic and subsequent reduction of in-person visits was assessed. This date represented the time at which the Australian government restricted numbers allowed to gather publicly and coincided with the first week in which funding was approved for hospital outpatient reviews to be conducted by telehealth for all patients.^14^

Data were collected from multiple sources. Electronic medical records (EMR) were searched for records of investigations completed at the CFSC or other public health institutes. The EMR was also searched for scanned and uploaded copies of external results. In addition, if the CFSC kept paper files for each patient, the files were manually searched to find paper records of any investigations undertaken. The Australian Cystic Fibrosis Data Registry (ACFDR) was also used as a data source to report on patient demographics, modulator use, total number of appointments per patient as well as the mode of outpatient care delivery, that is, in-person or telehealth (videocall or telephone call).

Ethical approval was obtained through the Sydney Children’s Hospitals Network Human Research Ethics Committee (2020/ETH03137).

### Study outcomes

The Australian Cystic Fibrosis Standards of Care^4^ were utilized to create a list of recommended annual investigations. The recommendations for the investigations within the Standards of Care differ in frequency, which is largely determined by patient age and clinical status. To allow valid comparison over two 12-month periods, analysis was limited to only the investigations within the Standards of Care which are recommended to be performed annually (Table 1).

**Table 1.** List of investigations recommended to be completed annually.

Investigation	Age group
CXR and/or chest CT scan	All ages
Blood tests, including Fat soluble vitamin levels (A, E, D), FBC, urea, electrolytes and creatinine (UEC), liver function tests (LFT)	All ages
Random or fasting blood glucose level (BSL)	All ages
Total IgE	All ages
Blood glucose monitoring by formal OGTT or fasting and random BSL on a regular basis	⩾10 years
Assessment of exercise tolerance using tests as appropriate to patient status and clinical standards	⩾6 years*
Sputum culture including screening NTM	All ages

Abbreviations: BSL, blood sugar level; CT, computerized tomography; CXR, chest X-ray; FBC, full blood count; IgE, immunoglobulin E; LFT, liver function tests; NTM, non-tuberculous mycobacteria; OGTT, oral glucose tolerance test; UEC, urea, electrolytes and creatinine.

* The minimum age for exercise testing was deemed to be 6 years of age as per current practice at the pediatric centers in the study.

To be considered in the analysis for each individual investigation, the investigation needed to be a recommended part of the assessment for that patient in both of the 12-month periods. For example, if a patient had an abnormal OGTT and was diagnosed with CFRD in the pre-pandemic 12-months, then they would not be recommended to have a OGTT in the following 12-months, therefore they would be excluded from the OGTT item analysis.

### Statistical analysis

Data were entered into SPSS Statistics for Windows version 28.0.0 (SPSS Inc., Chicago, Ill., USA). Descriptive statistics are presented in relation to demographics, clinic visit information and percentages of patients receiving recommendations in each 12-month period. McNemar’s test was used to compare the difference in frequency of recommended investigations completed in the 12-month periods before and after pandemic onset, with a significance level between pre and post pandemic frequencies set at 0.05.

## Results

A total of 625 patients received the entirety of their care at one of the 4 CFSCs during the data collection period and were eligible for inclusion in the analysis (Table 2).

**Table 2.** Demographics and characteristics of participants at commencement of the data collection period (16/3/2019).

Age, mean (SD)	19.8 (14.8)
Pediatric/Adult, n	310/315
Gender, M/F (n)	315/310
FEV1 % predicted entire cohort, mean (SD)	76.4 (24.5)
< 18	89.3 (17.7)
⩾18 years	68.3 (24.8)
BMI kg/m^2^ (participants aged ⩾17 years), mean (SD)	20.0 (4.1)
BMI *z*-score (participants aged 2 to <17 years), mean (SD)	0.01 (0.83)
Highly effective modulator use, n (%)	
Ivacaftor	43 (6.9%)
Elexacaftor/Tezacaftor/Ivacaftor	5 (0.8%)
Other modulator use, n (%)	
Lumacaftor/Ivacaftor	119 (19.0%)
Tezacaftor/Ivacaftor	20 (3.2%)

Abbreviations: BMI, Body Mass Index; FEV1, forced expiratory volume in one second.

### Mode of outpatient care delivery

Pre-pandemic onset, care was delivered primarily in-person, with all 4 centers having in-person visits in ⩾97% of cases. Prior to the pandemic, all centers aimed to complete annual investigations at a dedicated visit. There was no change in the median number of visits per patient before and after pandemic onset, however, following pandemic onset, all centers experienced a reduction of in-person visits and an increase in telehealth use. There was marked variation between CFSC models of care (Table 3), with Center 3 conducting in-person visits on 16% of occasions over the 12-month period, compared to 74% of all visits being in-person at Center 1.

**Table 3.** CF clinic reviews by type in the 12-months pre and post onset of the COVID19 pandemic.

	Center 1		Center 2		Center 3		Center 4	
	Pre	Post	Pre	Post	Pre	Post	Pre	Post
Clinic reviews per person (median, IQR)	5 (3-6)	5 (3-5)	4 (3-5)	4 (3-5)	4 (3-6)	5 (3-7)	5 (3-6)	5 (3-7)
Total reviews	846	795	545	561	670	729	1204	1314
Mode of review								
In-person, n (%)	846 (100%)	585 (74%)	542 (99%)	264 (47%)	650 (97%)	116 (16%)	1199 (99%)	898 (68%)
Telehealth, n								
Videocall	0	209	2	292	4	262	0	404
Telephone	0	0	1	5	16	351	5	12
Total, n (%)	0 (0%)	209 (26%)	3 (1%)	266 (53%)	20 (3%)	613 (84%)	5 (1%)	416 (32%)

### Frequency of annual investigations pre-pandemic

Pre-Pandemic Onset: There was variation in the frequency at which each CFSC performed annual screening investigations. Blood tests were performed with the highest frequency, with all centers collecting blood for full blood count/urea, electrolytes and creatinine/liver function tests (FBC/UEC/LFTs) in >80% of patients. Blood tests for Immunoglobulin E, blood sugar level (IgE, BSL) and vitamin levels were completed in >80% of patients at 3 out of 4 centers, however, 1 CFSC performed these tests less frequently. CXR was completed with the greatest consistency, with centers performing CXR in 73%-76% of patients. Completion of OGTT, exercise testing and annual sputum culture screening specifically for NTM exhibited the greatest variation between CFSCs with a range of 38%-69%, 3%-51%, and 53%-81% respectively. The recommended investigation performed least often at each CFSC prior to the pandemic was exercise testing (Table 4).

**Table 4.** Investigations completed in individual patients eligible for each investigation in the 12-months pre and post onset of the COVID19 pandemic.

	Center 1			Center 2			Center 3			Center 4		
	Pre	Post	*p*	Pre	Post	*p*	Pre	Post	*p*	Pre	Post	*p*
CXR or chest CT	131/179 (75)	132/179 (75)	.889	101/133 (76)	44/133 (33)	<.001	109/145 (75)	57/145 (39)	<.001	122/168 (73)	102/168 (61)	.023
Vitamins	164/179 (94)	155/179 (89)	.137	108/133 (81)	110/133 (83)	.625	134/145 (92)	112/145 (77)	<.001	112/168 (67)	97/168 (58)	.101
FBC/UEC/LFT	165/179 (92)	157/179 (88)	.170	109/133 (82)	111/133 (84)	.625	141/145 (97)	108/145 (74)	<.001	151/168 (90) 151/168	143/168 (85) 147/168	.201
Total IgE	163/179 (91)	156/179 (87)	.265	108/133 (81)	110/133 (83)	.625	137/145 (94)	111/145 (77)	<.001	124/168 (74)	115/168 (69)	.350
Random or Fasting BSL	161/179 (90)	155/179 (87)	.377	108/133 (81)	110/133 (83)	.625	137/145 (94)	108/145 (74)	<.001	96/168 (57)	67/168 (40)	<.001
OGTT	49/72 (68)	47/72 (65)	.891	44/80 (55)	23/80 (29)	<.001	63/101 (62)	41/101 (41)	.024	56/145 (39)	27/145 (19)	<.001
Exercise Test	60/115 (52)	32/115 (28)	<.001	27/100 (27)	8/100 (8)	.003	5/145 (3)	3/145 (2)	.453	41/168 (24)	23/168 (14)	.006
NTM screening	93/179 (52)	96/179 (55)	.787	57/133 (43)	54/133 (41)	.701	117/145 (81)	69/145 (48)	<.001	82/168 (49)	72/168 (43)	.261

Abbreviations: BSL, blood sugar level; CT, computerized tomography; CXR, chest X-ray; FBC, full blood count; IgE, Immunoglobulin E; LFT, liver function tests; NTM, non-tuberculous mycobacteria; OGTT, oral glucose tolerance test; UEC, urea, electrolytes and creatinine.

All results presented as n (%). The denominator indicates how many participants were eligible for each investigation in both 12-month periods.

### Frequency of annual investigations post-pandemic

In the 12 months following the pandemic onset, there was an overall reduction in annual screening investigations performed. Exercise testing, annual CXR and screening for CFRD using OGTT were most commonly reduced following pandemic onset, with 3 out of 4 centers showing a statistically significant decrease in testing rates for these assessments. Annual screening for NTM in sputum and investigations which involved a single blood collection (FBC/UEC/LFT, IgE, vitamin levels, and random/fasting BSL) continued at similar rates in the period following the pandemic onset, with 3 out of 4 centers having no statistically significant reduction in frequency of testing (Table 4).

There were between center differences in maintaining frequency of investigations post-pandemic onset. CFSC 1 demonstrated a statistically significant reduction in frequency of exercise testing only. CFSCs 2, 3, and 4 demonstrated significant (*P* < .05) reductions in 3, 7, and 4 of the 8 recommended investigations respectively (Table 4).

## Discussion

This study has shown that, despite the existence of National Standards of Care guidelines for managing CF, there was variability between CFSCs in performing recommended annual investigations before the onset of the COVID19 pandemic; and this variability was exacerbated during the pandemic. Blood tests continued with the greatest frequency, whilst other investigations which required attendance at the CFSCs, for example exercise testing, were most reduced. There were also variations in the mode of care delivery, with wide variation in the return to in-person visits between the different centers.

Across the 4 centers in the current study, there was variability in completion of recommended screening investigations pre-pandemic, with differing levels of variability dependent on the specific investigation. Investigations with the highest frequency of completion and least variation in the 12 months pre-pandemic were CXRs and blood tests. All 4 centers completed CXRs in approx. 75% of patients. Three out of 4 centers performed all recommended annual bloods in greater than 80% of patients. Whilst there is no published Australian data regarding completion of annual investigations, our study findings are consistent with the Annual Data Report from the CF Foundation Patient Registry (CFFPR) in the USA, which reports annual screening for fat soluble vitamins and liver enzymes in the pre-pandemic 12-month period occurring at a median (range) of 92% (72-100) and 92% (75-100), respectively.^15^

Investigations with high levels of variability between centers included screening for CFRD with OGTT and annual screening for NTM in sputum. OGTT screening in the current study ranged from 38% to 69% pre-pandemic. Data from the CFFPR also showed variation in completion of OGTT for the same time period, with OGTT performed at a median (range) of 61% (0%-100%) and 29% (0%-85%) in pediatric and adult centers respectively. Annual rate of NTM testing ranged from 43% to 81% across the 4 centers in the current study. Data from the CFFPR also showed variation in screening for NTM pre-pandemic, reporting a median (range) of 78% (27%-100%) across CF centers.^15^

The current study also demonstrated a reduced completion of annual screening investigations in the year following the onset of the pandemic. The pattern of reduction was inconsistent across the 4 centers in the current study. CFSC 1 had a statistically significant reduction in exercise testing alone, while CFSC 3 experienced a reduction in all investigations apart from exercise testing, however, the testing rate at CFSC 3 was very low both pre and post pandemic onset. Collaco et al^16^ analyzed CFFPR data to determine the extent to which persons with CF received certain components of recommended care in 2019versus 2020. There was minimal overlap in the recommendations analyzed by Collaco et al and those reported in the current study, with OGTT being the only investigation reported by both studies. Across all centers contributing data to CFFPR, Collaco et al reported OGTT rates declined from 63% to 48% in children and 33% to 19% in adults. Combining pediatric center data in the current study, the findings were very similar, with before and after pandemic OGTT testing rates of 62% and 48%, respectively. Combined adult data showed a reduction in OGTT completion from 48% to 28%.

Diseases other than CF which also have their care centered around specialist institutions and follow recommended standards of care have likewise reported reductions in investigations screening for complications completed during the COVID pandemic. In a multi-center survey of Italian inflammatory bowel disease services, Saibeni et al^17^ reported clear variations in how services modified their activity, with maintenance of biologic therapies delivered and a vast reduction in surveillance endoscopies and ultrasounds. There were also between center differences in care delivery to people with multiple sclerosis, with a reduction in number of recommended surveillance tests, including non-urgent MRIs and blood tests, being performed during the pandemic compared to a corresponding period pre-pandemic.^18,19^ Therefore, whilst there is limited comparable CF literature, wider evidence suggests disruption in maintaining selected services with a reprioritization of care.

The balance of in-person care versus telehealth appointments (telephone or audio-visual) was highly variable between the 4 centers in the current study. In-person visits over the first 12 months of the pandemic ranged from 16% to 74%, despite the four centers all being located in one major city. Dowd et al^12^ reported variation in frequency of in-person versus telehealth consultations over a wide geographical region of 286 North American centers. Six months into the pandemic, median in-person visit rate amongst centers was 57% with a wide IQR of 25%-86%. By 12 months into the pandemic, median in-person visits had increased to a median (IQR) of 80% (64%-95%).

The data from the current study suggests a link between return to in-person visits and completion of annual investigations. Center 1 returned to the highest percentage of in-person visits (74%) and had a statistically significant reduction in only 1/8 annual investigations. Center 3 had the lowest return to in-person visits (16%) and had a statistically significant reduction in 7/8 annual investigations. Whilst causation cannot be determined given the retrospective nature of the study, an association between return to in-person care and completion of recommended investigations is evident.

The variation in model of care delivery across the 4 CFSCs may be due to multiple reasons. Firstly, local health districts in conjunction with hospital executive units were independently responsible for decisions regarding access to outpatient services during the pandemic. Consequently, there was no uniform approach for access to CF services between the different centers. For example, one of the centers was only allowed to conduct in-person reviews for patients with symptoms of an acute exacerbation in the first 12 months of the pandemic, and subsequent to that, in-person reviews were limited to 25% of total clinic attendees (personal communication). Additionally, one of the centers was within the local health district responsible for the intake and medical management of returned overseas travelers positive for COVID19 requiring hospitalization or admission to Special Health Accommodation,^20^ which led to increased staffing pressures and reallocation of duties for members of the CF team.

There are limitations to this study. Firstly, clear reasons for variations in recommended investigations performed at each center were not documented and therefore unable to be reported. Therefore, only potential reasons for between center differences can be presented. For example, the health workforce at each CFSC may have been impacted to different degrees, with staff absences, furloughing, and redeployment, all having the potential to impact service delivery and accessibility of patients to the care team.^21,22^ Whilst not directly measured in this study, variable access to the CF care team has been reported in the USA.^23,24^ A second limitation was the reliance on medical records for capture of investigations performed. Whilst every effort was made to maximize validity of results such as searching of electronic medical records, scanned results from external facilities and hand searching of paper clinic files, it is possible that investigations were performed and not included in the study results.

Finally, the included investigations do not represent a comprehensive report of all recommended annual monitoring in patients with CF. For example, DEXA scans are recommended in adults and adolescents every 1 to 3 years, making any valid assessment for when a scan is due difficult to ascertain and therefore include within the 12-month time periods compared in this study. Annual assessments by allied health professionals including specific content, such as a musculoskeletal assessment and continence screening by a physiotherapist or dietary intake assessment by dietetics staff were also not included within the scope of this project which focused only on investigations.

In conclusion, the findings of this study show that even within one major city, there are marked variations in monitoring for complications of CF. In addition, the COVID19 pandemic led to reductions in completion of recommended investigations and further variation in care. The study emphasizes the need for CF specialist centers to audit their adherence to recommended standards of care along with research into methods for increasing adherence to standards, such as standardization of processes between centers. Ongoing evaluation is particularly important in light of emerging therapies and the changing model of care being delivered to patients with CF.
